# GPCRdb in 2025: adding odorant receptors, data mapper, structure similarity search and models of physiological ligand complexes

**DOI:** 10.1093/nar/gkae1065

**Published:** 2024-11-18

**Authors:** Luis P Taracena Herrera, Søren N Andreassen, Jimmy Caroli, Ismael Rodríguez-Espigares, Ali A Kermani, György M Keserű, Albert J Kooistra, Gáspár Pándy-Szekeres, David E Gloriam

**Affiliations:** Department of Drug Design and Pharmacology, University of Copenhagen, Universitetsparken 2, 2100 Copenhagen, Denmark; Department of Drug Design and Pharmacology, University of Copenhagen, Universitetsparken 2, 2100 Copenhagen, Denmark; Department of Drug Design and Pharmacology, University of Copenhagen, Universitetsparken 2, 2100 Copenhagen, Denmark; Department of Drug Design and Pharmacology, University of Copenhagen, Universitetsparken 2, 2100 Copenhagen, Denmark; Department of Structural Biology, St. Jude Children’s Research Hospital, 262 Danny Thomas Place, Memphis, TN 38105-3678, USA; Medicinal Chemistry Research Group, HUN-REN Research Center for Natural Sciences, Magyar tudósok körútja 2., Budapest H-1117, Hungary; Department of Drug Design and Pharmacology, University of Copenhagen, Universitetsparken 2, 2100 Copenhagen, Denmark; Department of Drug Design and Pharmacology, University of Copenhagen, Universitetsparken 2, 2100 Copenhagen, Denmark; Medicinal Chemistry Research Group, HUN-REN Research Center for Natural Sciences, Magyar tudósok körútja 2., Budapest H-1117, Hungary; Department of Drug Design and Pharmacology, University of Copenhagen, Universitetsparken 2, 2100 Copenhagen, Denmark

## Abstract

G protein-coupled receptors (GPCRs) are membrane-spanning transducers mediating the actions of numerous physiological ligands and drugs. The GPCR database GPCRdb supports a large global research community with reference data, analysis, visualization, experiment design and dissemination. Here, we describe our sixth major GPCRdb release starting with an overview of all resources for receptors and ligands. As a major addition, all ∼400 human odorant receptors and their orthologs in major model organisms can now be studied across the various data and tool resources. For the first time, a Data mapper page enables users to map their own data onto receptors visualized as a GPCRome wheel, tree, clusters, list or heatmap. The structure model data have been expanded with models of physiological ligand complexes and updated with new state-specific structure models of all human GPCRs (built using AlphaFold, RoseTTAFold and AlphaFold-Multistate). Furthermore, a structure or model (pdb file) can now be queried against GPCRdb’s entire structure/model collection through a *Structuresimilarity search* page implementing FoldSeek. Finally, for ligands, new search tools can query names, database identifiers, similarities or substructures against integrated entries from the ChEMBL, Guide to Pharmacology, PDSP K_i_, PubChem, DrugCentral and DrugBank databases. GPCRdb is available at https://gpcrdb.org.

## Graphical Abstract

**Graphical Abstract:**
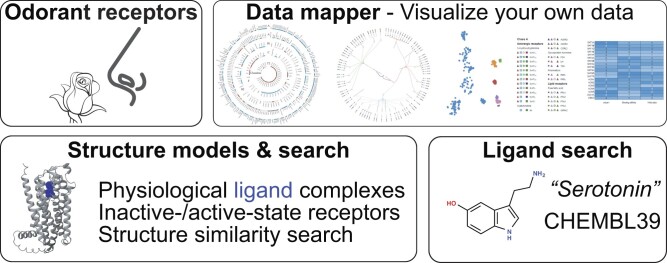

## Introduction

G protein-coupled receptors (GPCRs) make up 4% of the human genes (1), transduce the signals of two-thirds of physiological ligands (2) and mediate the actions of 34% of pharmaceutical drugs (3). The GPCR database GPCRdb serves a large research community worldwide with open access online resources for reference data, analysis, visualization, experiment design and data deposition. Since it was rebuilt in new form and migrated to the University of Copenhagen in 2014, GPCRdb has evolved over five major published updates (4–8) and recently has been complemented by GproteinDb (9,10), ArrestinDb (11) and Biased Signaling Atlas (12) dedicated to transducer proteins and ligand-dependent signaling bias, respectively, of GPCRs.

Over half of the human GPCR superfamily consists of ∼400 odorant receptors (ORs) that group into 18 subfamilies, which have formed the basis for the classification of the vertebrate OR superfamily (13). ORs have direct commercial applications across the perfume and food industries and have recently gained increased attention because of COVID19-related smell/taste impairment. Furthermore, several ORs have been found to be expressed outside the olfactory bulb or linked to physiological or pathogenic processes other than olfaction (14–16)—making it of fundamental importance to uncover their wider physiological functions and disease relevance. The major databases for ORs are ORDB and HORDE, but their latest release versions are from 2015 and 2019, respectively (17). More recent databases collate OR ligands/odors and their receptor interactions from experiments in OlfactionBase (18) and M2OR (19) or from predictions in Pred-O3 (20).

The known physiological ligands of GPCRs span 347 peptide/protein and 138 small molecule ligands forming 668 peptide/protein and 335 receptor complexes, respectively. These ligands are curated by the Nomenclature Committee of the International Union of Pharmacology, supplied in their database, Guide to Pharmacology (21), and can also be browsed in the *Physiological* ligands page in GPCRdb. Crystal and electron microscopy (cryo-EM) structures revealing the binding modes in ligand–receptor complexes are essential to understand the molecular mechanisms of ligand recognition and receptor activation, and also form the basis for deriving drugs through structure-based design. Unfortunately, such receptor complexes are still only available for 84 physiological ligands, but recent advances in molecular modeling software make it possible to generate models with higher accuracy and speed. Especially, AlphaFold-Multimer (22) and the RoseTTAFold all-atom protocol (23) open up for better modeling of peptide/protein and small molecule complexes, respectively [the AlphaFold 3 update also supports small molecules but has restricted usage (24)]. The impact of the former on GPCR–ligand complex modeling was clear from the last (2021) round of the global competition, ‘GPCR Dock’, in which many of the best models were generated with the help of AlphaFold-Multimer (25).

The structural coverage of GPCRs currently amounts to 200 distinct receptors, whereof 103 and 209 are in an inactive or active state, respectively (see *Structure* coverage page in GPCRdb). With respect to models, the 2023 release of GPCRdb (8) migrated from our in-house homology modeling pipeline (6,7) to AlphaFold-MultiState (26) to provide inactive- and active-state models of the human GPCRome (at the time, excluding ORs). These supplemented the single-state AlphaFold 2 models available from the European Bioinformatics Institute (27). Together, the many GPCR structures and models thereof open new opportunities for protein similarities to be assessed based on three-dimensional structural folds rather than primary sequences. As structure is more conserved than sequence (28), an advantage of this is higher sensitivity. Several structural alignment tools have been developed over the years, e.g. Dali (29), TM-align (30) and CE (31), but have been relatively slow hampering searches of many queries or large databases. However, in 2023 the Foldseek method and server gained wide popularity by increasing the speed four to five times (32).

In this article, we describe the 2025 GPCRdb release implementing the above-mentioned recent computational methods. The new data feature the addition of ORs and structure models of physiological ligand–GPCR complexes, as well as updated inactive-/active-state receptor models. The new tools span a Data mapper (user data mapped onto receptor plots) and powerful searches for ligands and similar structures. This further expands the utility of GPCRdb enabling scientific studies and structure-based drug design.

## Materials and methods

### Odorant receptor incorporation

We obtained the list of human ORs from the Human Genome Nomenclature Committee (https://genenames.org) (33) by filtering the Olfactory receptors gene group for entries with an assigned UniProt accession code. Pseudogenes were excluded. Species orthologs were retrieved from UniProtKB (34) by querying ‘Olfactory receptor’, excluding human entries and further filtering using the keywords ‘Olfaction’ and ‘Transmembrane helix’. We limited the species list to the most popular model organisms: mouse, rat, bovine, dog, rhesus monkey and chimpanzee and receptors that can be mapped to a human ortholog. For classification, we adopted the unified nomenclature for vertebrate olfactory receptors (13). Generic numbers, most specifically, the reference (X.50) positions, were inferred from a pairwise sequence alignment with the best hit from BLAST search against 25 ORs that had previously been included in GPCRdb and assigned generic numbers in the same way, but using non-odorant class A GPCR templates. The start and end of secondary structure segments were determined with DSSP (35,36) and custom segment definitions on AlphaFold models downloaded from the AlphaFold Protein Structure Database (https://alphafold.ebi.ac.uk). Where the pairwise sequence alignment produced misalignments, we manually edited segment start and end positions and the reference residue position (x50) for generic residue numbering (37).

### Data mapper development

Each plot has been developed and customized using either the D3 javascript library (tree, heatmap, dial and list) or the Plotly javascript library (38) (cluster). In the cluster plot, spatial positioning is computed using the *t*-distributed stochastic neighbor embedding (*t*-SNE) method (39) while the clusters are determined using *K*-means. Both calculations are performed using the scikit-learn Python module (40). Moreover, the default spatial positioning of clusters is derived from a pre-computed matrix of pairwise receptor similarities across the transmembrane helices 1–7 and helix 8. However, users can provide their own data type for the mapping (in the ‘Spatial positioning’ column in the data template for uploads). In this case, a pairwise distance matrix is computed on the fly and *t*-SNE is used to perform dimensionality reduction. *K*-Means is subsequently applied to assign the clusters.

### Modeling physiological ligand–GPCR–G protein complexes

Physiological ligands and their receptors were retrieved from GPCRdb (*Physiological* ligands page). For each receptor, the primary transducer G protein was retrieved from GproteinDb (*Couplings* page). The G protein subtype was chosen based on an activity measure (in order of preference), log(*E*_max_/EC_50_), efficacy, *E*_constitutive_ or activation rate (10). If subtypes had not been measured, we used the first G protein subtypes in the primary transducer family imported from the Guide to Pharmacology database (21). Small molecule and peptide/protein ligand complexes were modeled with the RoseTTA all-atom protocol and AlphaFold 2, respectively, applying the scoring shown in Supplementary Table S1. AlphaFold 2-based models (peptide/protein ligands) were built including the primary transducer G protein, whereas this was not computationally feasible for the RoseTTA all-atom protocol-based modeling of small molecule complexes.

For AlphaFold 2, we selected the model of each receptor and state with the lowest predicted aligned error (PAE) score. Additionally, we assessed the relative positioning of the peptide or protein ligand within the GPCR by selecting the models with the lowest PAE mean score (22). For the RoseTTA all-atom protocol, we applied a post-modeling Amber relaxation to improve the geometry and resolve steric clashes. The model quality was assessed through PAE mean of the seven transmembrane helices (7TM PAE mean), with a cutoff value of max 10. The accuracy of the small molecule positioning within the binding site was also evaluated by the predicted local distance difference test (pLDDT) mean score, with a cutoff threshold of 60, where higher scores were considered acceptable (23).

### Modeling inactive- and active-state receptors

We modeled all human GPCRs in their inactive and active states based on structural templates released in the Protein Data Bank (41) by May 2024. Initially, we employed the standard AlphaFold 2 protocol with all available templates, without distinguishing between activation states. The best resulting models were then classified as either inactive or active as in (42). The missing state was then modeled based on state-filtered templates using AlphaFold-Multistate (26), as we did in (8).

### Structure similarity search implementation

The new page *Structuresimilarity search* implements Foldseek (32). The structure similarity search functionality was implemented as a Django (43) view. To manage Foldseek’s execution, a containerization approach using Docker was employed. The Docker SDK (44) for Python was used to control Docker containers programmatically from within the Django view. We generated a custom Foldseek Docker image, developed Django view methods for handling user requests, prepared input data, executed Foldseek within containers and processed results. The view also includes methods to retrieve and integrate additional structure information from the database.

### Ligand database update and search tool development

The previous GPCRdb release (8) had implemented a ligand matching functionality enabling integration of several external drug [DrugBank (45) and DrugCentral (46)] and ligand databases [ChEMBL (47), Guide to Pharmacology (21) and PubChem (48)]. To resolve duplicate ligand entries, we added the information regarding HELM notation for peptide ligands where available, thus minimizing the possibility of redundant entries based on name or amino acidic sequence.

We equipped the page *Ligands(by ligand query)* with extensive search functionality featuring new types of input queries, matching algorithms and batch searches. SMILES and SMARTS representation and matching were implemented based on the open-source cheminformatics software RDKit (https://www.rdkit.org). Molecular similarity, substructure and exact match search were developed based on RDKit’s PostgreSQL cartridge (version 2024_03_3) and the django-rdkit library [snapshot version 2023-05-3 (https://github.com/rdkit/django-rdkit)]. The latter implements the exact structure search operator from the RDKit PostgreSQL cartridge for SMILES exact match. For SMARTS, exact structure search was implemented as a substructure search, but restricting the results only to structures with matching atomic degrees (number of bonds or connections) for any kind of atom in the query SMILES. This was done using the RDKit PostgreSQL cartridge *mol_adjust_query_properties* function for adjusting the query properties (query parameters or options). Molecular similarity was computed from the RDKit implementation of Morgan 2 fingerprints (two-dimensional), which are ECFP (extended-connectivity fingerprint)-like fingerprints (49,50), using the default radius of two bonds, the default fingerprint size of 2048 bits and Tanimoto similarity ratio (51) as similarity value. The chemical feature definitions used to generate the fingerprints were adapted from (52).

## Results

### Odorant receptors

**Figure 1.**
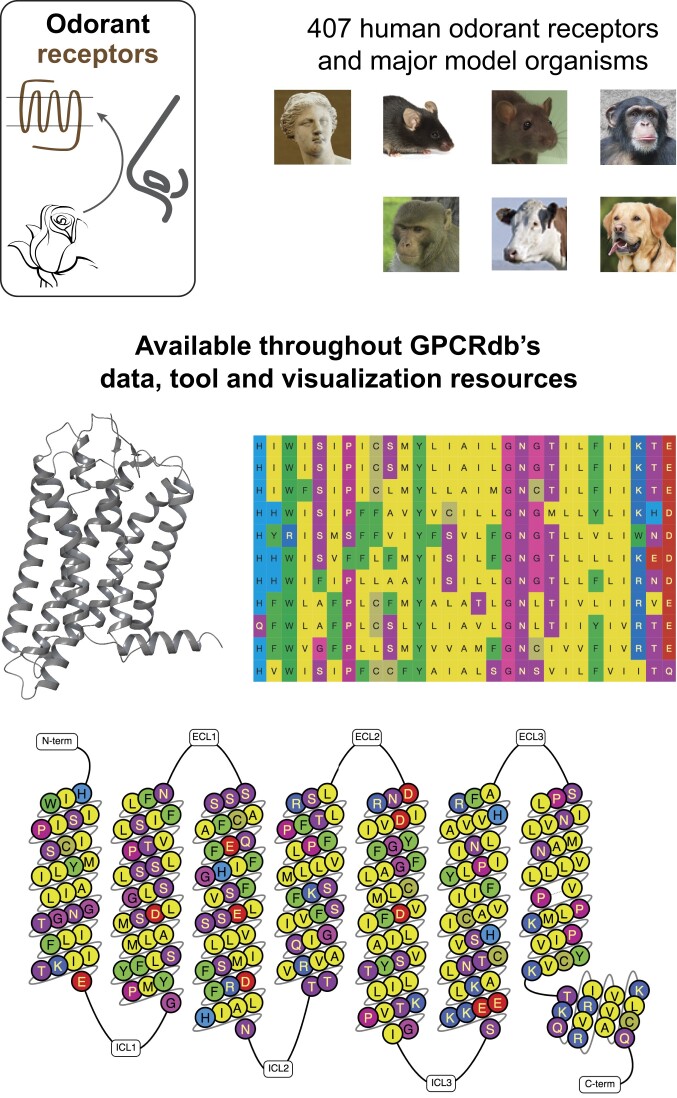
ORs. All 407 human ORs and orthologs in major model organisms are now available throughout GPCRdb’s data and tool resources, here exemplified by a structure model, sequence alignments and snake plot.

This release of GPCRdb adds all 407 human ORs available from the Human Genome Nomenclature Committee (genenames.org) (33) and orthologs from UniProtKB (34) covering popular model organisms: mouse, rat, bovine, dog, rhesus monkey and chimpanzee. These receptors span two classes: Class O1 (fish-like odorant) and Class O2 (tetrapod-specific) with families 51, 52, 56 and 1–14, respectively. Class O1 contains 57 while Class O2 has 350 of the human receptors. All of these receptors are now available across all of GPCRdb’s data and tool resources (Figure 1).

### Data mapper

Data mapper is a new section of GPCRdb that lets users upload data for any set of human receptors to swiftly map them using several visualization options suitable for e.g. publication or presentation. The Data mapper tool guides users from a main page to a data overview and the plotting interface pages. The main page provides a data template (MS Excel) for upload that features a dedicated tab for each available plot, with columns for each online data point and dropdown menus to choose between numerical or categorial data types. Once the user data has been uploaded, a data overview pop up shows errors, if any, in the data formatting. After this check, the Data mapper redirects to a plotting page with a tab for each plot for which the user uploaded data.

**Figure 2.**
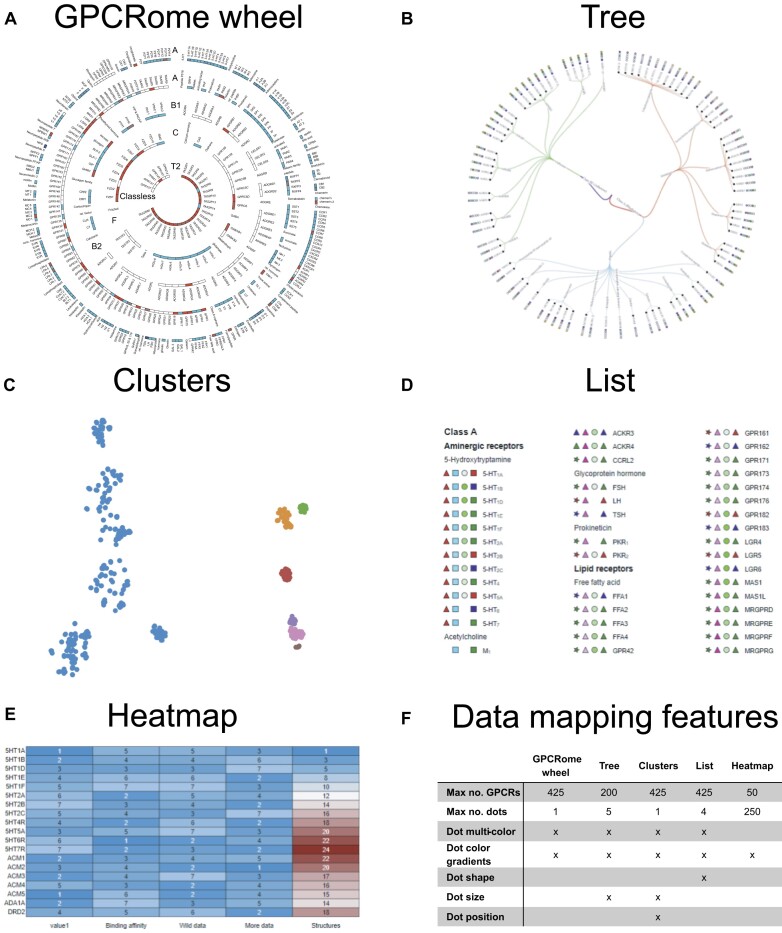
Data mapper plots and features. The new Data mapper section allows users to upload and map their own data onto five different receptor plots. (**A**) The GPCRome wheel shows all human non-odorant GPCRs in concentric circles containing one or two GPCR classes and alphabetically arranging receptors by their family (by physiological ligand) and name. (**B**) The Tree shows only the user-uploaded receptors classified by class, ligand type and receptor family. (**C**) The Clusters group receptors based on their sequence similarity or user-supplied data. (**D**) The List provides a space-efficient enumeration GPCRs by classification and (**E**) the Heatmap colors cells by user data. Panel (**F**) summarizes the customizable mapping features for each plot.

While the five featured plots differ in their layout structure, they all share dedicated control panels aimed at enhancing the available customization of the data visualization. These allow binary, discrete/categorial and continuous values to be visualized with different colors (black/white, grayscale or multi-color) or shapes (circle, star, triangle, square and diamond) (see Figure 2F). Other options adjust layout and formatting of the overall plot, such as spacing, font size and line thickness. All plots can be downloaded in vector (svg) or bitmap file (png, jpeg and tiff) formats.

*GPCRome wheel* (Figure 2A, ∼400 GPCRs). The GPCRome wheel plot spans the non-odorant human GPCRome in a single data mapping visualization. The outer to inner layers list receptors by their class, as follows: class A with known physiological ligands, class A orphans (have unknown physiological ligands), class B (B1/secretin and B2/adhesion families), class C, class T2 and class F. In each class, receptors are listed alphabetically, first by their receptor family (not shown) and secondly by the receptor name. Data are mapped to a circular band divided into sections next to the name of the receptors for the subset of those that was uploaded by the user. Other receptors not uploaded by the user stay shown in the plot to put the receptors with data into context of the overall GPCRome. This is useful when showing the coverage of data, for example, which receptors have or are missing an experimental structure. For this reason, the GPCRome wheel is now also used in the pages *Structure* coverage, *Ligand coverage* and *Mutation coverage—*replacing the previous classification trees.

*Tree* (Figure 2B, ≤200 GPCRs). The Tree plot shows (only) the receptors uploaded by the user arranged in a classification tree that branches outward on the levels of classes, ligand type (e.g. aminergic, lipid or peptide), receptor family and receptor. Data can be mapped in an internal dot shown at the end of the branch, and in up to five external dots that are placed outside of the receptor name.

*Clusters* (Figure 2C, ≤50 GPCRs, if no/little overlap). The Cluster plot clusters user-uploaded receptors based on their sequence similarity across their shared segments, the transmembrane helices 1–7 and helix 8. Alternatively, users can replace the sequence similarity to instead cluster receptors based on own data type, which is entered in the ‘Spatial positioning’ column in the data template for uploads. The number of clusters can be adjusted to group receptors into fewer, larger or more, smaller clusters. The data points can be alternative shapes, and their color can be by cluster (default) or defined in the data upload.

*List* (Figure 2D, ∼400 GPCRs). The List plot lists receptors grouped by headings for the class, ligand type and/or receptor family (default is set to class and receptor family). Uniquely for this plot, the grouping can be changed by omitting any of the classification levels. Data are mapped to up to four dots, or other shapes (circle, star, triangle, square and diamond). The user has the option to change the layout (number and size of columns), text-styling for each category separately, and dynamically change the coloring of continuous data input.

*Heatmap* (Figure 2E, ≤50 GPCRs, if single-column figure). The Heatmap plot maps data to cells (max 250) instead of dots. The cells highlight data by applying a color gradient (default from blue to red) based on the user-supplied values. The heatmap can be styled in various ways to best fit the user’s preference by moving or rotating the data labels, showing/hidden borders or data values, or resizing the font size of receptors, data labels or data values.

### Structure models of physiological ligand complexes

The previous publication of GPCRdb added a *Physiological* ligands page (8) presenting all 1003 known human physiological ligand–receptor pairs, and 275 from non-human species, available from the Guide to Pharmacology database (21). Of these pairs, only 84 (8%) have experimental structures and these are available from the *Structures* page. Therefore, this release adds models of physiological ligands in complex with their receptors and, for peptide/protein ligands, also the primary transducer G proteins (all human). These models are provided in a new *Structure* models (ligand complexes) page tabulating information about all models, including the ligand name and type (small molecule, peptide or protein) as well as receptor name and classification. Furthermore, both models and experimental structures are also provided in a new section of the existing page for *Physiologicalligands*.

**Figure 3.**
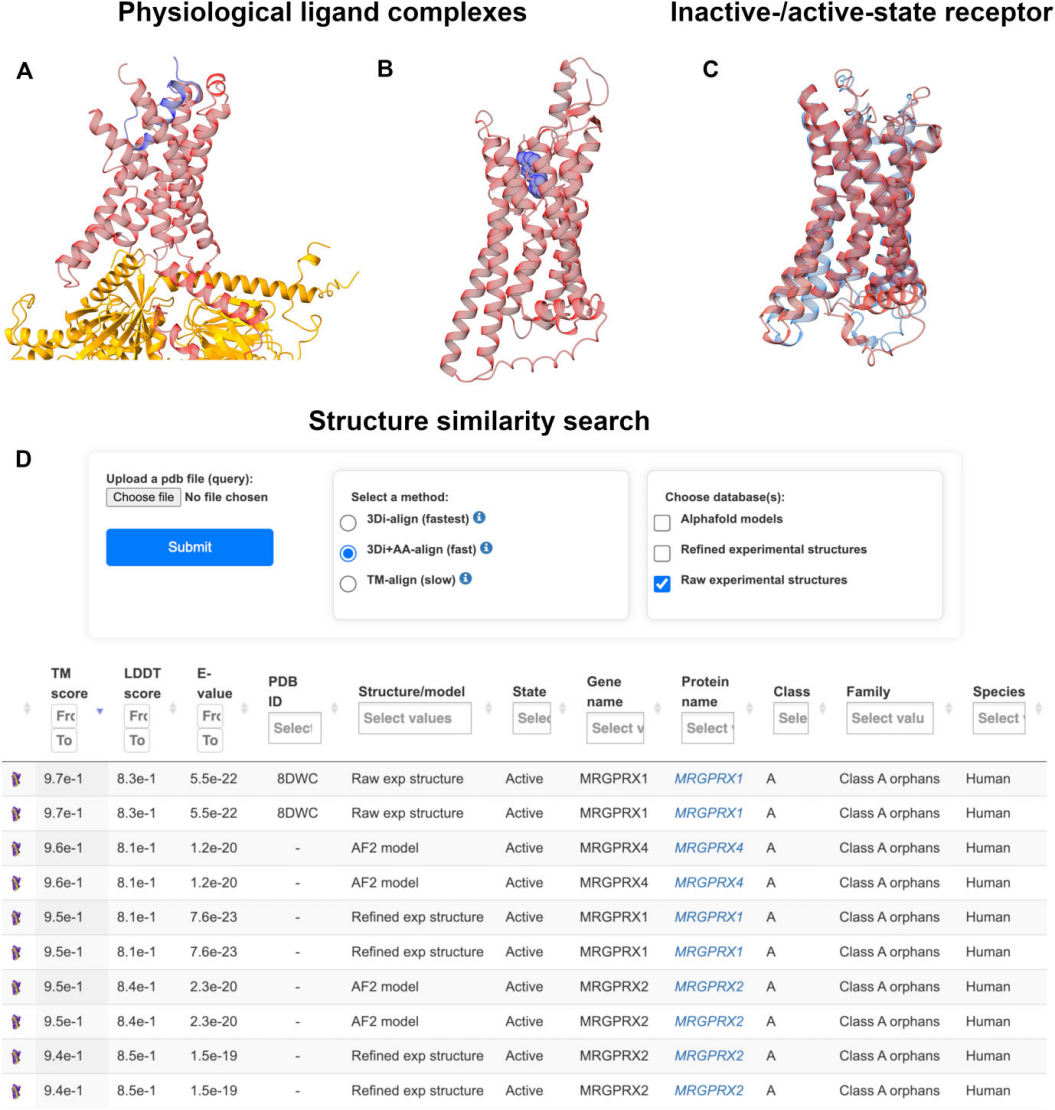
New structure models and similarity search. (**A**) Example of a model of a physiological peptide ligand in complex with its receptor and a G protein (amylin, calcitonin receptor and G_s_) built with AlphaFold 2. (**B**) Example of a model of a physiological small molecule ligand in complex with its receptor and a G protein (adenosine, adenosine receptor A1) built with the RosettaFold all-atom protocol. (**C**) Example of inactive and active states models (of the eicosatetraenoic acid receptor) built with AlphaFold-Multistate. (**D**) The most similar experimental structures and models of human GPCRs after querying MRGPRX1 in the *Structure* similarity search page, which is based on the integration of the Foldseek algorithm.

After applying stringent quality criteria (‘Materials and methods’ section and Supplementary Table S1), these pages currently present models of 465 peptide/protein ligand and 87 physiological small molecule ligand complexes, as exemplified in Figure 3A and B. Together with the experimental structures, these cover 507 (76%) and 129 (38%) of all peptide/protein and small molecule ligand complexes, respectively, with GPCRs. This provides an, although not complete, unprecedented coverage and accessibility of physiological receptor–ligand interactions serving as a structural basis for a range of computational and experimental studies.

Each model is further described in an individual page. Here, a 3D viewer maps the pLDDT score, which is the confidence level for each residue, onto the structure model of the proteins. Next to the structure view, horizontal bars provide several scores within defined ranges (min and max). For peptide/protein ligand models, built using AlphaFold-Multimer, the additional scores are PAE mean (ligand–receptor), pTM (global complex) and ipTM (interfaces of ligand–receptor–G protein complex). For small molecule ligand models built using the RoseTTA all-atom protocol, additional bars display the scores 7TM PAE mean (overall receptor quality) and small molecule pLDDT mean (accuracy of ligand positioning in the binding site). Together, these scores cover individual residues, molecules and complexes—providing the information necessary to evaluate the structure models facilitating appropriate usage in structure-guided research.

### Updated inactive-/active-state receptor models

The *Structure* models (receptor) page now avails inactive-active state structure models of all human GPCRs—including 814 OR models, (exemplified in Figure 3C). Compared to the previous release (8), the updated models are based on a larger pool of experimental templates. A first set of models, generated using AlphaFold 2, are based on all templates available by May 2024 and cover one activation state of each receptor (unless it was intermediate state and filtered out). This is the case for 41 active-state and 244 inactive-state models. The second set of models, built by AlphaFold-Multistate, then cover the missing inactive or active state of each receptor based on the respective subset of templates. In this way, a maximum number of templates have been used while generating both receptor states. Compared to other resources, GPCRdb also has a larger coverage of receptors. This release includes 97 receptors (97 inactive-state models and 97 active-state models) not available in the AlphaFold-Multistate archive (last updated in 2021). Furthermore, unlike the AlphaFold Protein Structure Database (last updated in 2022), our release provides both states for each receptor, offering a more complete structural representation.

### Structure similarity search

The new page *Structure* similarity search implements Foldseek (32) to search a user-uploaded query (pdb, cif or mmcif file), which could be a structure or model from GPCRdb or any resource. The search database encompasses all receptor structures and/or models in GPCRdb. The pairwise similarity assessment focuses on the transmembrane helices 1–7 that are shared by all GPCRs, while leaving out segments with larger conformational variability and lower confidence. The resulting top hits are sorted by decreasing the template modeling (TM) score that measures global structural similarity (53). They can be re-sorted by LDDT score (local distance difference test) or e-values measuring local residue accuracy and statistical significance of the alignment, respectively.

The *Structuresimilarity search* page offers three structural alignment methods depending on the use case. For rapid local structural comparisons, the 3Di-align algorithm is recommended. This method efficiently encodes tertiary interactions using the 3Di alphabet representation. For improved accuracy in local alignments, the 3Di + AA-align method is preferred. It enhances the 3Di approach by incorporating amino acid substitution scores, albeit with increased computational requirements. For detecting fold similarities across diverse protein families, the TM-align procedure is advised. This method excels in global structural alignments through its implementation of a TM-score rotation matrix and dynamic programming, despite its higher computational cost compared to the other approaches.

### Ligand search

**Figure 4.**
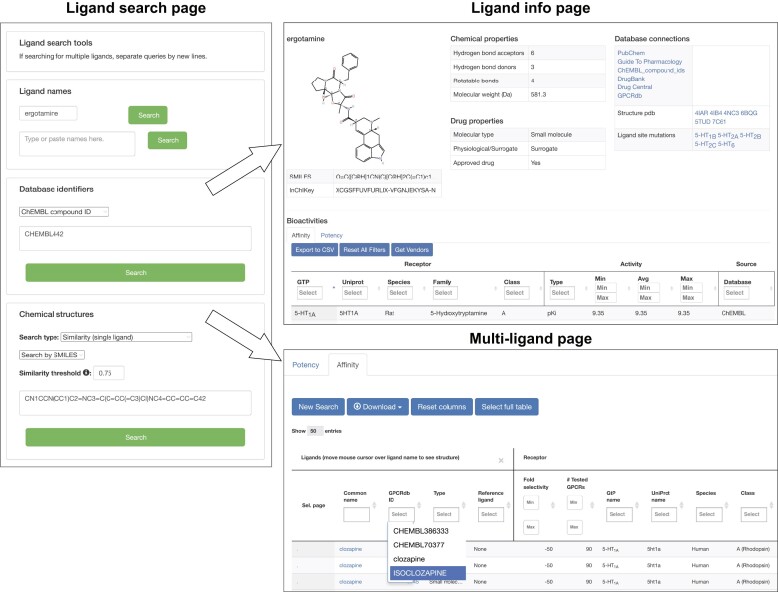
Ligand search tool and results pages. Left: the page *Ligands(by ligand query)*now support querying of ligand names, database identifiers and structures. Top: the ligand info page shows the result for searches matching an individual ligand, here ergotamine springing from a query of the ChEMBL identifier CHEMBL442. Bottom: the ligand browser shows the results for searches matching multiple ligands. Here, the results represent ligands with a Tanimoto coefficient ≥0.75 compared to the query, the SMILES of clozapine.

GPCRdb integrates GPCR ligands and bioactivities from the ChEMBL (47), Guide to Pharmacology (21) and PDSP K_i_ (https://pdsp.unc.edu/databases/kidb.php) databases. Here, the page *Ligands(by ligand query)* has been equipped with extensive search functionality (Figure 4, left):

- Ligand name searches feature matching to aliases and autocompletion listing alternative ligand entries—thereby facilitating finding of ligands while navigating similar entries.
- Ligand database identifier lookup, spanning ChEMBL, Guide to Pharmacology, DrugBank (45), DrugCentral (46) and PubChem (48) identifiers.
- Ligand structures in the form of SMILES or SMARTS can be queried for exact match, substructure search or similarity search.
- Ligand structures as Standard InChiKeys (54) can be queried for an exact match.

Single ligand results redirect to a detailed ligand info page described in (8) and shown in Figure 4 (top-right). This page shows biological activities (potency and affinity) per receptor, assay information and selected chemical properties (molecular weight, number of hydrogen bond acceptors and donors and rotational bonds and LogP). Multi-ligand results (from similarity, substructure or structural exact match search, name search or batch database identifier lookup) are tabulated in a ligand browser (Figure 4, bottom). This browser contains the resulting ligands’ name, with a small pop-up showing a ligand structure representation; GPCRdb ID, key features from the above (and a link to) ligand info page and aggregated biological activity data.

## Discussion

With the introduction of Data mapper, GPCRdb has gained the ability to visualize data coming from users. It can support a wide range of use cases, as it can be used for diverse data types, without expertise in data analytics software and across scientific publication, presentation and teaching. The heatmap, cluster, list and tree plots are well-known visualizations for which the main benefit will be the increased accessibility and swiftness of figure production. Uniquely, the GPCRome plot offers a new solution to mapping the entire human non-odorant GPCRome in a single, one-page image. It replaces an iconic image in the GPCR community, a phylogenetic tree of the human GPCR families published in 2003 (55) for example, often used for to flag crystal or cryo-EM structures. It has the advantages of giving an up-to-date listing of receptors (none missing) and classification into classes/families, for example class T2 (Taste 2 receptors) forming its own class rather than joint with the class F (Frizzled family) (56). Furthermore, also receptors without mapped data stay shown (in gray font) putting the highlighted receptors into context of the overall GPCRome and informing which receptors are missing the given type of data. In GPCRdb, the GPCRome plot has been added to the pages *Structure coverage, Ligand coverage* and *Mutation coverage*.

As a future perspective, we plan to adapt the Data mapper to map data stored in GPCRdb and develop data mapping plots for residues. The snake plot and helix box plots are already shown in the *Receptor* page, where they can be colored manually, but do not support data upload.

Advances in structure modeling have previously led GPCRdb to replace its own in-house homology modeling pipeline (6,7) with AlphaFold-MultiState (26). Furthermore, GproteinDb and ArrestinDb provide models of G protein and arrestin complexes, respectively, built using AlphaFold-Multimer (10,11,22). Here, this trend continues for physiological ligand complexes using AlphaFold-Multimer and RoseTTAFold. For peptide/protein ligand complexes, we included also a G protein as that ensures that the ligand is not incorrectly modeled into the intracellular-facing G protein pocket. Having a complete model of an activated GPCR, i.e. both agonist- and G protein-bound receptor, allows a more accurate modeling of the receptor outside of the ligand pocket and also makes the system suitable for molecular dynamics simulations, including those investigating ligand interactions (temporally). Whereas we could build high-confidence models for most (465 out of 668) peptide/protein ligand–receptor pairs using AlphaFold-Multimer, our first implementation of RoseTTAFold could only model roughly one-third (87 out of 335) of small molecule–receptor complexes with adequate scores (‘Materials and methods’ section and Supplementary Table S1). A lower success rate is expected, as modeling of small molecule complexes is more challenging and recent than that of peptide/protein ligands. It will be very interesting to see to what extent AlphaFold 3 (24), or future updates of the RoseTTAFold all-atom protocol, can cover more of the small molecule-activated receptor systems.

The addition of a new type of data often allows it to be used together with pre-existing tools and vice versa. This is the case for ORs (data) and the new tools for ligand search and receptor structure similarity search presented herein. Whereas other sensory receptors (for e.g. taste and vision) have long been included, the ORs were left out because of their larger numbers (∼400 in human) and, historically, an uncertain gene repertoire due to species-specific duplication and pseudogenization. Now any researcher can study them on par with other receptors across resources, such as sequence alignments, structures and structure models [for odorants/ligands, we refer to other databases (18–20)]. Conversely, the receptor structure similarity search is a new tool that ties directly into big datasets already stored in GPCRdb. It can search structures, refined structures (8) and structure models individually or combined. The focus on transmembrane helices 1–7, leaving out the N-/C-termini and loops, facilitates comparison on diverse or incomplete receptors. Finally, the ligand search provides a multi-type entry portal to the ∼220 000 ligands already aggregated in GPCRdb from the ChEMBL (47), Guide to Pharmacology (21), PDSP K_i_ (https://pdsp.unc.edu/databases/kidb.php), DrugBank (45), DrugCentral (46) and PubChem (48) databases. Whereas these ligands could previously only be accessed by their name, indirectly via the receptor target name, the database identifier and structural searches are new, as well as the ability to query multiple ligands simultaneously.

## Supplementary Material

gkae1065_Supplemental_File

## Data Availability

GPCRdb is available at https://gpcrdb.org and can also be accessed via a RESTful API, which complies with the OpenAPI specification using Swagger (code examples are available at https://docs.gpcrdb.org/web_services.html). The source code, the underlying data and a virtual machine configuration are all available in the repositories at https://github.com/protwis. The source code is also available in Zenodo at https://doi.org/10.5281/zenodo.14041766 and the data at https://doi.org/10.5281/zenodo.14041749.
