# Supplementary material for: GPCRdb in 2025: adding odorant receptors, data mapper, structure similarity search and models of physiological ligand complexes
Source: Nucleic Acids Res. 2024 Nov 18;53(D1):D425–35. doi: 10.1093/nar/gkae1065 (PMC11701689; doi:10.1093/nar/gkae1065)
Supplement: gkae1065_Supplemental_File [file gkae1065_supplemental_file.pdf]

Supplementary Table 1. Scores shown for AlphaFold2 and RoseTTA models of peptide/protein and small molecule ligands, respectively in complex with their receptor.

Method	Score	Comparison	Ligand	GPCR	G protein	Confidence assessment	Cut-off
**Both**	pLDDT	Within each protein	x	x	x	Placement of each residue relative to its preceding and succeeding residues.	(1)
**AlphaFold**	PAE mean	Ligand relative receptor	x	x		Relative position of ligand and receptor.	Lowest (1)
**AlphaFold**	pTM	Global complex	x	x	x	Overall fold and topology.	0.5 (2)
**AlphaFold**	ipTM	Proteins interfaces	x	x	x	Relative positioning of protein chains at the interface.	0.5 (2)
**RoseTTA**	pLDDT mean	Ligand atoms to ligand/receptor atoms <15 Å	x	x		Placement of ligand atom relative to all other atoms within 15 Å.	60 (3)
**RoseTTA**	7TM PAE mean	Within receptor		x		Relative positioning of residue pairs in the receptor 7TM domain.	10 (3)

References

1. Tunyasuvunakool, K., Adler, J., Wu, Z., Green, T., Zielinski, M., Zidek, A., Bridgland, A., Cowie, A., Meyer, C., Laydon, A. *et al.* (2021) Highly accurate protein structure prediction for the human proteome. *Nature*, **596**, 590-596.
2. Xu, J. and Zhang, Y. (2010) How significant is a protein structure similarity with TM-score = 0.5? *Bioinformatics*, **26**, 889-895.
3. Krishna, R., Wang, J., Ahern, W., Sturmfels, P., Venkatesh, P., Kalvet, I., Lee, G.R., Morey-Burrows, F.S., Anishchenko, I., Humphreys, I.R. *et al.* (2024) Generalized biomolecular modeling and design with RoseTTAFold All-Atom. *Science*, **384**, eadl2528.
